# Genetic Characterization of Multidrug-Resistant *Acinetobacter baumannii* and Synergy Assessment of Antimicrobial Combinations

**DOI:** 10.3390/antibiotics13111079

**Published:** 2024-11-13

**Authors:** Aurora Luna-De-Alba, Samantha Flores-Treviño, Adrián Camacho-Ortiz, Juan Francisco Contreras-Cordero, Paola Bocanegra-Ibarias

**Affiliations:** 1 Laboratory of Immunology and Virology, School of Biological Sciences, Autonomous University of Nuevo Leon, Monterrey 66455, Nuevo Leon, Mexico; maria.lunad@uanl.edu.mx (A.L.-D.-A.); contrerasjfco@gmail.com (J.F.C.-C.); 2 Department of Infectious Diseases, University Hospital Dr. José Eleuterio González, Autonomous University of Nuevo Leon, Monterrey 64460, Nuevo Leon, Mexico; samflorest@gmail.com (S.F.-T.); acamacho_md@yahoo.com (A.C.-O.)

**Keywords:** genetic characterization, colistin, meropenem, efflux pumps, porins

## Abstract

**Background/Objectives**: *A. baumannii* is a prominent nosocomial pathogen due to its drug-resistant phenotype, representing a public health problem. In this study, the aim was to determine the effect of different antimicrobial combinations against selected multidrug-resistant (MDR) or extensive drug-resistant (XDR) isolates of *A. baumannii*. **Methods**: MDR or XDR *A. baumannii* isolates were characterized by assessing genes associated with drug resistance, efflux pumps, porin expression, and biofilm formation. The activities of antimicrobial combinations including tigecycline, ampicillin/sulbactam, meropenem, levofloxacin, and colistin were evaluated using checkerboard and time-to-kill assays on isolates with different susceptibility profiles and genetic characteristics. **Results**: Genetic characterization of MDR/XDR strains (*n* = 100) included analysis of OXA-24/40 gene carbapenemase (98%), genes encoding aminoglycoside-modifying enzymes (44%), and *parC* gene mutations (10%). AdeIJK, AdeABC, and AdeFGH efflux pumps were overexpressed in 17–34% of isolates. Omp33-36, OmpA, and CarO membrane porins were under-expressed in 50–76% of isolates; CarO was overexpressed in 22% of isolates. Isolates showed low biofilm production (11%). Synergistic activity was observed with levofloxacin-ampicillin/sulbactam and meropenem-colistin, which were able to inhibit bacterial growth. **Conclusions**: Genetic characteristics of *A. baumannii* were highly variable among the strains. Synergistic activity was observed with meropenem-colistin and levofloxacin-ampicillin/sulbactam combinations in the checkerboard method, but not in the time-to-kill assays. These discrepancies among both methods indicate that further studies are needed to determine the best therapeutic combination for treating infections by *A. baumannii*.

## 1. Introduction

*Acinetobacter baumannii* is a Gram-negative coccobacillus associated with several hospital-acquired infections, occurring in critically ill patients, such as ventilator-associated pneumonia and bacteremia, with attributable mortality rates up to 35% [1]. This pathogen represents a worldwide public health problem due to its ability to survive on different surfaces of the hospital environment, and its ability to acquire and develop a diversity of antimicrobial resistance mechanisms against different antibiotics [2]. Whilst carbapenems are considered the first choice of treatment against *A. baumannii* infections, the relentlessly increasing prevalence of carbapenem-resistant *A. baumannii* (CRAB) strains signifies a threat to susceptible patients, increasing mortality up to 70% [1,3,4].

Given the rising rates of resistance to multiple antimicrobials and the lack of development of new molecules with efficacy against this pathogen, the antibiotic combination therapy has been considered as a strategy to effectively control *A. baumannii* infection [5]. The antibiotic combination therapy uses two or more drugs with different mechanisms of action to treat a bacterial infection, in order to improve therapeutic efficacy, delaying the development of drug resistance, reducing toxicity, and broadening the spectrum of antibacterial activity [6].

Although there is still no consensus on the optimal treatment of CRAB infections, colistin is most often used in combination with other antibiotics, such as carbapenems, fosfomycin, tigecycline, ampicillin/sulbactam, vancomycin, or rifampin [1]. However, resistance to colistin can occur in up to 30% of CRAB strains, complicating the treatment of CRAB infections [1,3].

Evaluating the in vitro activity of antimicrobial combinations against a bacterial pathogen is challenging due to the technically complex and time-consuming process. Some of the most used techniques to assess the in vitro activity of antimicrobial combinations are the checkerboard and time-to-kill assays. In this study, the aim was to determine the effect of different antimicrobial combinations against selected multidrug-resistant (MDR) or extensive drug-resistant (XDR) isolates of *A. baumannii*.

## 2. Results

### 2.1. Characteristics of MDR and XDR Isolates

During the two-year period, 263 *A. baumannii* clinical isolates were collected from 192 patients. Patients were predominantly hospitalized in the intensive care unit (55.7%, *n* = 107), in the COVID unit (24.5%, *n* = 47), in the internal medicine ward (15.1%, *n* = 29), and other medical wards (4.7%, *n* = 9).

Out of the 192 strains, 91.7% (*n* = 176) were either MDR (78.6%, *n* = 151) or XDR (13.1%, *n* = 25), of which 90.1% (*n* = 173) were CRAB isolates. Out of the 176 strains classified as either MDR or XDR, 100 isolates were obtained from respiratory tract specimens and were further selected for genetic characterization and synergy effect testing. These selected isolates presented high resistance to ceftazidime (100%), levofloxacin (100%), imipenem (98%), meropenem (98%), piperacillin/tazobactam (97%), cefepime (95%), and gentamicin (89%). Lower rates of resistance to tigecycline (65%), ampicillin/sulbactam (35%), and colistin (1%) were detected.

### 2.2. Genetic Characteristics of MDR and XDR Isolates 

Regarding carbapenemases, 98% (*n* = 98) of the resistant strains carried the *OXA-24/40* gene (a class D carbapenemase) and 100% (*n* = 100) the *OXA-51* gene (a species-specific intrinsic carbapenemase). *KPC*, *VIM*, *IMP*, *NDM*, and *mcr* genes were not detected in any of the isolates. Genes encoding aminoglycoside-modifying enzymes were distributed heterogeneously among the isolates. At least one gene was detected in 44% of the strains, of which the most frequent was *aph*(3′)VIa (31%), followed by *ant*(2′)Ia (25%), *aph*(3′)IIa (12%), and *aac*(6)Ib (12%). In 53.2% (50/94) of gentamicin non-susceptible isolates, no genes encoding aminoglycoside-modifying enzymes were detected. Up to 10% of the isolates presented mutations in *parC* gene associated with fluoroquinolone resistance. No mutations were detected in *gyrA*, *pmrA,* and *pmrB* genes, associated with quinolone and polymyxin resistance.

**Figure 1:**
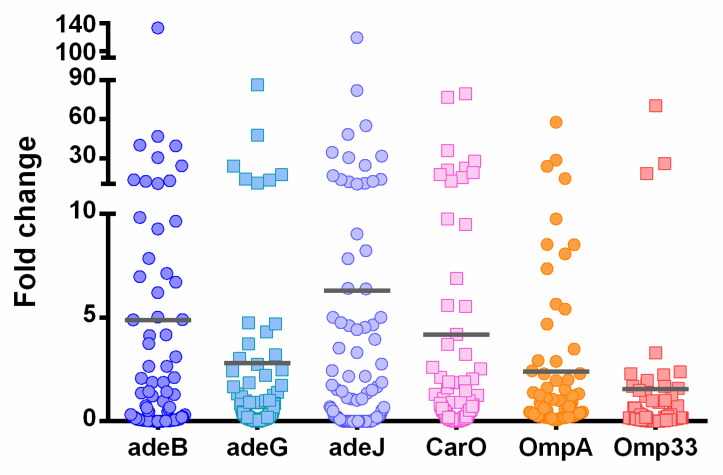
Expression levels of efflux pumps and membrane porins in MDR *A. baumannii* strains. The expression levels of AdeABC, AdeFGH, and AdeIJK pumps and CarO, OmpA, and Omp33-36 membrane porins are shown, compared to the reference strain (*A. baumannii* ATCC 17978), used as baseline. The line represents the mean of each fold change.

In addition, efflux pump overexpression was observed in 34% of the isolates for AdeIJK (2.2–119.3-fold change), 29% for AdeABC (2.1–133.3-fold change), and 17% for AdeFGH (2.2–86.4-fold change). Membrane porins were under-expressed (<0.5 fold) in most isolates, 76% for Omp33-36, 54% for OmpA, and 50% for CarO, although 22% of isolates showed CarO overexpression (Figure 1). Regarding biofilm production, 11% (*n* = 11) of the isolates were biofilm producers, 6% presented high biofilm production, and 5% were low biofilm producers.

### 2.3. Activity of Antimicrobial Combinations by the Checkerboard Assay

Isolates were first classified into groups according to their antimicrobial resistance profile and genetic characteristics. However, the majority of isolates presented a unique pattern, and categorization was not possible. Consequently, 42 strains were randomly selected to evaluate the synergistic effects of antimicrobial combinations (Table S1). A heterogeneous behavior was observed among the isolates after exposure of combined tested antibiotics (Table 1). Although most of the isolates showed indifferent activity to several antimicrobial combinations (38.1–97.6%), some isolates presented additive activity to levofloxacin-SAM (28.6%), meropenem-colistin (19.0%), colistin-levofloxacin (14.3%), tigecycline-colistin (2.4%), and meropenem-levofloxacin (2.4%). Antagonistic activity was observed for meropenem-colistin (40.5%), tigecycline-levofloxacin (9.5%), meropenem-levofloxacin (4.8%), levofloxacin-SAM (2.4%), and tigecycline-meropenem (2.4%).

Synergistic activity was observed with levofloxacin (16 µg/mL) and SAM (16/8 µg/mL) with FICI = 0.5 in one isolate (19-2211), and with meropenem (16 µg/mL) and colistin (1 µg/mL) with FICI = 0.3 in another isolate (20-0329), as shown in Table 2. The isolates in which the synergistic activity was observed showed different genetic characteristics. Isolate 19-2211 showed CarO overexpression and both OmpA and Omp33-36 under-expression. Isolate 20-0239 showed overexpression of adeFGH pump, OmpA, and Omp33-36. Neither isolate showed mutations associated with quinolone or polymyxin resistance, nor were they biofilm producers (Table S1).

### 2.4. Activity of Antimicrobial Combinations by the Time-to-Kill Method

**Figure 2:**
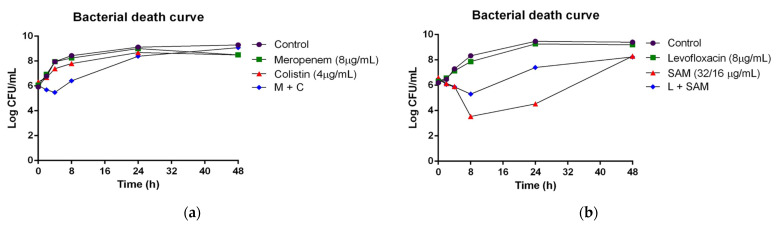
Time-to-kill curve of MDR/XDR *A. baumannii* isolates under the combination of two antibiotics. The time-to-kill curve per hour is shown for two different MDR/XDR *A. baumannii* isolates: (**a**) isolate 20-0329, treated with the combination of meropenem (16 µg/mL) and colistin (1 µg/mL), and (**b**) isolate 19-2211, treated with the combination of levofloxacin (16 µg/mL) and SAM (16/8 µg/mL). C: colistin, L: levofloxacin; M: meropenem; MDR: multidrug-resistant; SAM: ampicillin/sulbactam; XDR: extensive drug-resistant.

The bacterial inhibitory effect of synergistic antimicrobial concentrations was evaluated using time-to-kill curves. According to the results, both antimicrobial combinations (levofloxacin-SAM and meropenem-colistin) were able to partially inhibit bacterial growth (Figure 2). Particularly, the combination of meropenem and colistin caused a decrease in bacterial growth during the first 4 h, unlike the effect shown in each antibiotic individually. However, this antimicrobial effect remained the same regardless of single or dual combination after 24 h, which increased after 48 h of incubation. Bacterial regrowth was observed after 8 h of incubation with meropenem plus colistin. Instead, an antagonistic effect was observed with the combination of SAM and levofloxacin, although higher bacterial inhibition was observed after using SAM alone.

## 3. Discussion

Over the years, *A. baumannii* has emerged as a prominent nosocomial pathogen due to its MDR phenotype, representing a public health problem. Increased morbidity and mortality can be associated with MDR and XDR phenotypes. Our study shows that 78.6% of the resistant isolates exhibited an MDR profile, while 13.1% exhibited an XDR profile, and 90.1% were CRAB. Past studies from Mexico also showed lower MDR values (44.3%) compared to ours, although XDR values were similar in our strains compared to previous reports of 11.4–56.6% [7,8]. These results suggest a greater capacity for adaptation and dissemination of XDR strains, highlighting the importance of *A. baumannii* in the hospital setting and the need to research alternative therapy solutions, such as antimicrobial combination therapy. In this study, we evaluated the prevalence of *A. baumannii* drug resistance phenotypes and characterized their genetic characteristics to assess antibiotic combinations with specific synergistic activity.

The genetic characterization of the strains, particularly carbapenem resistance, showed OXA-24/40 in 98% of the strains and the species-intrinsic OXA-51 gene in all the strains. Compared to previous results [9], our results show increased OXA-24/40 frequency (98% vs. 25.7%) and decreased OXA-58 detection (0% vs. 28.3%). Regarding aminoglycoside resistance, *aph*(3′)VIa was the most frequent gene in non-susceptible isolates, although lower than other studies (52% vs. 31%) [10]. Regarding fluoroquinolone resistance, only 10% of the isolates presented mutations in *parC* gene. Overexpression of efflux pumps (AdeIJK, AdeABC, and AdeFGH) associated with the MDR phenotype was also detected, similar to previous studies [11]. Efflux pump substrate affinities and expression levels can be associated with different resistance to multiple antibiotics, e.g., AdeIJK has a broader substrate spectrum than AdeABC pump [12]. Membrane porins were all under-expressed, Omp33-36 more than OmpA and CarO. Omp33-36 loss is more common in pneumonia isolates, as is the majority of isolates analyzed in our study [13]. A low frequency of efflux pump overexpression is associated with CRAB [8], which in our study was 98%.

Biofilm formation promotes antibiotic resistance in *A. baumannii,* as transmission of resistance mechanisms occurs among bacterial strains within biofilms. Previous studies show that the prevalence of antimicrobial resistance correlated with strong biofilm formation, as higher biofilm production was observed for XDR strains compared to MDR strains [14]. In this study, low biofilm production was detected, differing from previous studies (11% vs. 90.8%) [8]. These results suggest that our MDR *A. baumannii* possesses diverse resistance mechanisms, which help the bacteria to adapt and survive in different environments.

In vitro activity of drug combinations on *A. baumannii* strains with different genetic characteristics was assessed by checkerboard and time-to-kill assays. Selected drugs were chosen based on their specific mechanism of action, e.g., tigecycline (protein synthesis), SAM and meropenem (cell wall synthesis), levofloxacin (DNA replication), and colistin (cell membrane permeability). Synergistic activity was observed only with two antimicrobial combinations, meropenem-colistin and levofloxacin-SAM, in two different strains.

Meropenem-colistin combination showed not only synergistic and additive effects, but mainly antagonistic activity, which differs from previous results [15], in which antagonism was not observed in doripenem. Doripenem was used instead of meropenem due to its stability against carbapenemases. However, we did not use doripenem due to its lack of availability in our country. Furthermore, the synergistic activity of meropenem-colistin occurred in a strain resistant to both antibiotics. As available options to treat carbapenem- and colistin-resistant *A. baumannii* are limited and in most cases empirical, our results provide insight regarding the use of meropenem-colistin. However, while meropenem-colistin caused a decrease in bacterial growth during 4 h, bacterial regrowth was observed after 8 h, which increased after 48 h. In a previous study [16], the activity of colistin against *Klebsiella pneumoniae* in 24 h time-to-kill assays also showed initial killing followed by regrowth of strains at 24 h, suggesting a bacteriostatic effect rather than bactericidal, as might be our case. Thus, further studies are needed to assess a greater strain sample to investigate whether the synergistic activity of meropenem-colistin is related to the antimicrobial susceptibility profile or genetic characteristics. Levofloxacin plus SAM was the combination with higher additive effect, and synergy was observed in one isolate. A previous study showed 90% of synergistic activity on isolates resistant to both levofloxacin and SAM [17], which suggests it might be considered a good therapeutic option, although further studies are needed to assess strains with more diverse mechanisms of resistance. Tigecycline, in three different combinations, presented predominantly indifferent activity and no synergistic activity. However, previous studies reported synergy of tigecycline and levofloxacin in 16.7% of isolates [18], and of tigecycline plus colistin in 40.6% of isolates [15].

High variability was observed in the genetic characteristics of the population studied, which may explain the overall low synergistic activity observed. Synergistic activity was observed in *A. baumannii* strains with different genetic characteristics and different colistin, gentamycin, and SAM susceptibility. Synergistic activity may be influenced by the variability in the mechanisms involved in bacterial drug resistance [19]. In previous studies, higher synergism was observed in isolates with high MIC values [19,20,21]. Likewise, in our study, a MIC reduction was observed for meropenem (64 vs. 16 µg/mL), colistin (16 vs. 1 µg/mL), and levofloxacin (64 vs. 16 µg/mL) after using antibiotic combinations in dual therapy. According to our results, dual therapy reduced the concentration of antibiotics needed to inhibit bacterial growth compared to monotherapy not only in isolates with synergistic activity, but also in those with additive and indifferent effects. These results suggest dual therapy could offer an advantage for clinical treatment; however, more in vitro and in vivo studies are required.

One limitation of this study is that time-to-kill curves using optimal concentrations from the checkerboard assay did not confirm the previously observed synergistic activity. Several factors, such as bacteria and drug type, drug concentration, exposure time, and analysis method may account for the discrepancy between the checkerboard and time-to-kill assays, which can show variable results [22]. Therefore, the selection of the most appropriate method and the validation of the results with complementary methods are important. In addition, the analysis of antibiotic combinations and concentrations not included in time-to-kill assays might allow one to decipher the dose dependency of the observed synergistic or antagonistic activity. Furthermore, none of the in vitro synergistic assays are standardized, and their results may be controversial; therefore, data should be analyzed with caution and should be correlated with the clinical data of the patient.

## 4. Materials and Methods

### 4.1. Study Population

Consecutive *A. baumannii* isolates were collected during 2019 and 2020 from the routine microbiology laboratory of the Dr. José Eleuterio González University Hospital, a tertiary-care teaching hospital with 600 hospital beds located in Monterrey, Mexico. The hospital has a yearly average of 25,000 hospitalizations, and it receives patients transferred from other regional hospitals and from the northeastern states of Mexico. Only one isolate per patient and from respiratory tract specimens (bronchial lavage, bronchoalveolar lavage, endotracheal aspirate, and expectoration) were selected for the study.

### 4.2. Culture and Identification of Clinical Isolates

The strains were grown on blood agar plates (BD Bioxon, Mexico City, Mexico) and incubated at 37 °C for 24 h. *Acinetobacter calcoaceticus-baumannii* complex identification was performed using matrix-assisted laser desorption ionization-time of flight mass spectrometry (MALDI-TOF MS, Microflex LT system, Bruker Daltonics, Bremen, Germany) as described by the manufacturer. The identification of *A. baumannii* species was performed by *recA* and 16S-23S rRNA intergenic spacer genes amplification using the primers and conditions described previously [23].

### 4.3. Antimicrobial Susceptibility Testing

Antimicrobial susceptibility testing was conducted by disk diffusion according to the recommended methods in the M100 and breakpoints established in M02 protocols of the Clinical and Laboratory Standards Institute (CLSI, Wayne, PA, USA) [24]. The antibiotics tested were ampicillin/sulbactam (SAM), piperacillin/tazobactam, cefepime, ceftazidime, imipenem, meropenem, gentamicin (GEN), levofloxacin, and tigecycline (Thermo Fisher Scientific Oxoid Ltd., Basingstoke, UK). Colistin screening was performed using the colistin broth disk elution and confirmation was evaluated by broth microdilution as recommended by the CLSI.

The isolates were classified as non-MDR, MDR, or XDR according to previous recommendations [25]. Isolates non-susceptible (either intermediate or resistant) to three or more antibiotic categories were considered as MDR. Isolates non-susceptible to at least one agent from all but two or fewer antibiotic categories were considered as XDR. Only MDR or XDR isolates were selected for further analysis.

### 4.4. Genetic Characterization

#### 4.4.1. Detection of Antimicrobial Resistance-Associated Genes

Either the presence or a mutation of different antimicrobial resistance-associated genes were evaluated by PCR and DNA sequencing. The carbapenemase-encoding genes analyzed were class A β-lactamases (*KPC*), metallo-β-lactamases (*IMP*, *VIM*, and *NDM*), and OXA-type (*OXA-23-like*, *OXA-24/40-like*, *OXA-51-like*, and *OXA-58-like*) using primers and conditions previously reported [26]. The genes encoding the following aminoglycoside-modifying enzymes were analyzed using the primers and conditions previously reported [27]: *aph*(3′)Ia, *aph*(3′)VIa, *aac*(3′)Ia, *aac*(3′)IIa, *acc*(6′)Ib, *aac*(6′)Ih, and *ant*(2′)Ia. Colistin resistance-associated mcr gene was also detected by PCR [28]. Mutations in *parC*, *gyrA*, *pmrA,* and *pmrB* genes were submitted for large-scale DNA sequencing (Macrogen, South Korea) using the primers and conditions suggested previously [29,30]. The sequences were analyzed on the BioEdit platform (Informer Technologies, Inc., Los Angeles, CA, USA). Mutations were searched using the reference strains *A. baumannii* (GenBank accession number X82165.1) for *gyrA* and (GenBank accession number X95819.1) for *parC*; *A. baumannii* strain AB67 (GenBank accession number MF673422.1) for *pmrA,* and *A. baumannii* strain S402 (GenBank accession number MK660501.1) for *pmrB*.

#### 4.4.2. Assessment of Efflux Pump and Porin Expression

The expression of *adeB*, *adeG,* and *adeJ* (genes belonging to efflux pump systems AdeABC, AdeFGH, and AdeIJK, respectively) and *ompA*, *carO,* and *omp33* (genes harboring porins or outer membrane proteins) was determined by RT-qPCR [31,32,33,34]. Total RNA was extracted from a 4−5 h log phase culture of *A. baumannii* using the RNeasy mini kit (Qiagen, Venlo, The Netherlands) according to the manufacturer’s instructions. RNA concentration and purity were determined using a NanoDrop spectrophotometer (ND-1000, Wilmington, NC, USA). Quantification of *adeB*, *adeJ*, *ompA*, *carO*, and *omp33* was performed using the SuperScript III platinum One-step qRT-PCR system (Invitrogen, Cergy Pontoise, France). *adeG* quantification was performed using the PowerUp SYBR Green Master Mix for qPCR (Applied biosystems, Foster, CA, USA). The RT-qPCR was performed using 20 ng of RNA and the primers and probes described in Supplementary Table S1 in a Bio-Rad CFX instrument (Bio-Rad, Hercules, CA, USA). *rpoB* gene was used as a housekeeping gene to normalize the expression of target genes [34]. The 2^−∆∆CT^ method was used to calculate the relative gene expression. Results were shown as the relative expression of the mRNA compared with that of *A. baumannii* ATCC 17978. Each experiment was performed in triplicate. A relative expression >2.0 and <0.5 were considered as overexpression and under-expression, respectively [35].

### 4.5. Assessment of Biofilm Formation

Semiquantitative determination of biofilm formation was performed by crystal violet staining as previously described, with some modifications such as no glucose supplementation to the broth [36]. The biofilm index (BI, ratio of optical density (OD) of biofilm cell to the OD of planktonic cells [ODbiofilm/ODplanktonic]) was used to normalize the amount of biofilm formed to the total cell content of each sample tested to avoid variations due to differences in bacterial growth. Biofilm production was classified according to the BI: non-producer (BI < 0.90), weak producer (BI = 0.90−1.20), and strong producer (BI > 1.20) as previously described [37]. *Staphylococcus aureus* ATCC 29213 (high biofilm producer) and *Escherichia coli* ATCC 25923 (low biofilm producer) were used as quality control strains.

### 4.6. Determination of Antibacterial Activity by Antimicrobial Combinations

Isolates were classified according to their susceptibility profile and genetic characteristics in order to evaluate the effect of different antibiotic combinations. Synergistic effects between tigecycline, SAM, meropenem, levofloxacin, and colistin were assessed for the selected isolates using the checkerboard microdilution method. The selection of antibiotics to be tested in combination was selected according to the mechanisms of action of each antibiotic and the pharmacological drug interactions between antibiotics. The combinations used were colistin-meropenem, colistin-levofloxacin, colistin-tigecycline, meropenem-levofloxacin, levofloxacin-SAM, tigecycline-levofloxacin, and tigecycline-meropenem [38].

#### 4.6.1. Checkerboard Method

A bacterial inoculum of 0.5 McFarland was 1:150 diluted in Mueller–Hinton broth, and 100 μL was transferred to 96-well round-bottom plates (Corning Inc., Corning, NY, USA) containing serial dilutions of antibiotics. The antibiotics used were colistin (0.12–2 µg/mL), meropenem (8–256 µg/mL), levofloxacin (2–64 µg/mL), tigecycline (2–64 µg/mL), and SAM (8/4–256/128 µg/mL). The plate was then incubated for 24 h at 37 °C. The fractional inhibitory concentration index (FICI) was calculated from the sum of the fractions of inhibitory concentrations. The results were categorized as synergism (FICI ≤ 0.5), additive (FICI > 0.5–1), indifferent (1 < FICI ≤ 4), and antagonism (FICI ≥ 4) [39]. 

#### 4.6.2. Time-to-Kill Method

Isolates, antibiotic combinations, and concentrations that showed best synergistic activity by the checkerboard test were further selected for analysis using time-to-kill assays. All assays were performed three times. A bacterial inoculum was tested against different antibiotics, individually or in combination with the previously selected concentrations. For the combinations which included levofloxacin, colistin, and sulbactam, an induction was previously carried out to express resistance to that specific antibiotic. An inoculum of 0.5 MacFarland from a 24 h bacterial culture was inoculated in a 15 mL tube containing the concentrations of levofloxacin-sulbactam and colistin-meropenem equivalent to the FICI demonstrating synergy by the checkerboard method. Cultures were incubated at 37 °C and 100 µL was obtained at 0, 2, 4, 8, 24, and 48 h after incubation, serially diluted in 0.9% saline, and transferred to trypticase soy plates to determine colony counts after incubation. Bactericidal activity was defined as a reduction of ≥3 log^10^ colony-forming unit (CFU)/mL compared to the initial inoculum after 24 h of exposure. A reduction of ≥2 log^10^ CFU/mL compared to the most active antimicrobial agent alone was considered as synergistic. An increase of ≥2 log^10^ CFU/mL compared to the most active antimicrobial agent alone was considered as antagonistic [40].

### 4.7. Statistical Analysis

Isolate classification was done using re-scaled distance cluster combination analysis in IBM SPSS Statistics 25 version. The graphs were created using IBM SPSS Statistics or GraphPad Prism (GraphPad Software Inc., San Diego, CA, USA) version 8.0.

## 5. Conclusions

Synergistic activity against MDR *A. baumannii* strains was observed with meropenem-colistin and levofloxacin-SAM combinations. However, the checkerboard and the time-to-kill assays showed discrepancies, indicating that further studies are needed to properly select the most appropriate method. Additionally, the variability of the genetic characteristics of *A. baumannii* strains might have influenced the low synergistic activity observed. In addition, the synergistic activity of meropenem-colistin occurred in a strain resistant to both antibiotics, and dual therapy reduced the concentration of antibiotics needed to inhibit bacterial growth compared to monotherapy. These results suggest dual therapy could offer an advantage for clinical treatment; however, more studies are needed to determine the best therapeutic combination for treating infections by *A. baumannii*.

## Figures and Tables

**Table 1:** Dual-therapy results for different antimicrobial combinations against MDR and XDR *A. baumannii* isolates.

Antimicrobial Combination *	No. (%) of Isolates with Combined Effect			
	Synergistic	Additive	Indifferent	Antagonistic
LEV + SAM	1 (2.4)	12 (28.6)	28 (66.7)	1 (2.4)
MEM + COL	1 (2.4)	8 (19.0)	16 (38.1)	17 (40.5)
TGC + MEM	0 (0.0)	0 (0.0)	41 (97.6)	1 (2.4)
TGC+ COL	0 (0.0)	1 (2.4)	41 (97.6)	0 (0.0)
TGC + LEV	0 (0.0)	0 (0.0)	38 (90.5)	4 (9.5)
MEM + LEV	0 (0.0)	1 (2.4)	39 (92.9)	2 (4.8)
COL + LEV	0 (0.0)	6 (14.3)	36 (85.7)	0 (0.0)

* SAM: ampicillin/sulbactam; MEM: meropenem; LEV: levofloxacin; TGC: tigecycline; COL: colistin.

**Table 2:** Comparison of concentrations used in monotherapy and dual therapy and antimicrobial activity.

Antimicrobial Combination *	Isolate	MIC of Individual Antibiotic (µg/mL)		MIC of Antibiotics in Combination (µg/mL)		FICI	Activity
		Antibiotic 1	Antibiotic 2	Antibiotic 1	Antibiotic 2		
LEV + SAM	19-2211	LEV (64)	SAM (16/8)	LEV (16)	SAM (16/8)	0.5	Synergistic
	20-0046	LEV (32)	SAM (16/8)	LEV (16)	SAM (32/16)	2.5	Indifferent
MEM + COL	20-0329	MEM (64)	COL (16)	MEM (16)	COL (1)	0.3	Synergistic
	19-0705	MEM (64)	COL (0.25)	MEM (256)	COL (0.25)	5.0	Antagonistic
TGC + MEM	19-0002	TGC (2)	MEM (64)	TGC (2)	MEM (8)	1.1	Indifferent
	19-0360	TGC (2)	MEM (64)	TGC (2)	MEM (8)	1.1	Indifferent
TGC+ COL	20-0008	TGC (2)	COL (1)	TGC (2)	COL (0.12)	1.1	Indifferent
	20-0327	TGC (2)	COL (0.5)	TGC (2)	COL (0.12)	1.2	Indifferent
TGC + LEV	19-0115	TGC (2)	LEV (32)	TGC (2)	LEV (2)	1.1	Indifferent
	20-0046	TGC (2)	LEV (2)	TGC (8)	LEV (8)	8.0	Antagonistic
MEM + LEV	20-0098	MEM (64)	LEV (4)	MEM (32)	LEV (16)	4.5	Antagonistic
	20-0406	MEM (64)	LEV (32)	MEM (32)	LEV (8)	0.8	Additive
COL + LEV	19-1092	COL (1)	LEV (16)	COL (0.25)	LEV (16)	1.3	Indifferent
	20-0048	COL (0.5)	LEV (16)	COL (0.25)	LEV (4)	0.8	Additive

* SAM: ampicillin/sulbactam; MEM: meropenem; LEV: levofloxacin; TGC: tigecycline; COL: colistin; FICI: fractional inhibitory concentration index.

## Data Availability

The original contributions presented in this study are included in the article and Supplementary Materials. Further inquiries can be directed to the corresponding author.

## Supplementary Materials

The following supporting information can be downloaded at: https://www.mdpi.com/article/10.3390/antibiotics13111079/s1, Table S1: Susceptibility profile and genetic characteristics of the *A. baumannii* isolates selected for the checkerboard assay.
