# Supplementary material for: Genetic Characterization of Multidrug-Resistant Acinetobacter baumannii and Synergy Assessment of Antimicrobial Combinations
Source: Antibiotics (Basel). 2024 Nov 13;13(11):1079. doi: 10.3390/antibiotics13111079 (PMC11591439; doi:10.3390/antibiotics13111079)
Supplement: Supplementary file 1 [file antibiotics-13-01079-s001.zip › antibiotics-3304810-supplementary.pdf]

Table S1. Susceptibility profile and resistome of the *A. baumannii* isolates selected for the checkerboard assay (n = 42)

Isolate	Antimicrobial resistance profile										Antimicrobial resistance mechanisms/resistome						
	SAM	TZP	FEP	CAZ	IMI	MEM	GEN	LEV	TGC	COL	Carbapenem resistance genes	Aminoglycoside-modifying genes	Quinolone resistance-associated mutations	Efflux pumps overexpression	Porins under-expression	Porins overexpres sion	Biofilm production
**19-0002**	S	R	R	R	R	R	S	R	R	I	OXA-24/40, OXA-51	Not detected	*parC* mutations	None	CarO, OmpA, Omp33-36	None	None
**19-0010**	R	R	R	R	R	R	R	R	R	I	OXA-24/40, OXA-51	Not detected	*parC* mutations	None	CarO, OmpA, Omp33-36	None	None
**19-0115**	S	R	R	R	R	R	R	R	R	I	OXA-24/40, OXA-51	Not detected	Not detected	AdeIJK	CarO, Omp33-36	None	None
**19-0255**	R	R	R	R	R	R	R	R	R	I	OXA-24/40, OXA-51	*aph*(3')IIa, *aac*(6')Ib	Not detected	AdeIJK	CarO, OmpA, Omp33-36	None	None
**19-0256**	R	R	R	R	R	R	R	R	S	I	OXA-24/40, OXA-51	*aph*(3')VIa, *ant*(2')Ia	Not detected	AdeIJK	CarO, OmpA	Omp33-36	None
**19-0360**	R	R	R	R	R	R	R	R	R	I	OXA-24/40, OXA-51	Not detected	Not detected	None	CarO, OmpA, Omp33-36	None	None
**19-0705**	S	R	R	R	R	R	R	R	R	I	OXA-24/40, OXA-51	Not detected	Not detected	AdeABC	OmpA, Omp33-36	CarO	None
**19-0823**	S	R	R	R	S	S	R	R	R	I	OXA-51	*aph*(3')IIa, *aac*(6')Ib	Not detected	None	CarO, OmpA, Omp33-36	None	None
**19-1084**	S	R	R	R	R	R	R	R	R	I	OXA-24/40, OXA-51	Not detected	Not detected	AdeIJK	CarO, OmpA	Omp33-36	None
**19-1092**	S	R	R	R	R	R	R	R	R	I	OXA-24/40, OXA-51	Not detected	Not detected	AdeABC	OmpA, Omp33-36	CarO	None
**19-1116**	R	R	R	R	R	R	R	R	S	I	OXA-24/40, OXA-51	*aph*(3')VIa, *ant*(2')Ia	Not detected	AdeFGH	Omp33-36	None	None
**19-1203**	R	R	R	R	R	R	R	R	S	I	OXA-24/40, OXA-51	*aph*(3')VIa, *ant*(2')Ia	*parC* mutations	None	CarO, OmpA, Omp33-36	None	None
**19-1455**	R	R	R	R	R	R	R	R	S	I	OXA-24/40, OXA-51	Not detected	Not detected	AdeIJK	CarO	OmpA	None
**19-1826**	S	R	R	R	R	R	S	R	R	I	OXA-24/40, OXA-51	Not detected	Not detected	AdeABC, AdeFGH, AdeIJK	Omp33-36	OmpA	None
**19-2055**	R	R	R	R	R	R	R	R	R	I	OXA-24/40, OXA-51	*aph*(3')VIa, *ant*(2')Ia	Not detected	AdeIJK	CarO, OmpA, Omp33-36	None	None
**19-2211**	R	R	R	R	R	R	R	R	R	I	OXA-24/40, OXA-51	*aph*(3')VIa, *ant*(2')Ia	Not detected	None	OmpA, Omp33-36	CarO	None
**19-2249**	R	R	R	R	R	R	R	R	R	I	OXA-24/40, OXA-51	*aph*(3')IIa, *aac*(6')Ib	Not detected	None	CarO, OmpA, Omp33-36	None	None
**19-2250**	R	R	R	R	R	R	R	R	R	I	OXA-24/40, OXA-51	*aph*(3')IIa, *aac*(6')Ib	Not detected	None	CarO, OmpA, Omp33-36	None	None
**20-0008**	S	R	R	R	R	R	R	R	R	I	OXA-24/40, OXA-51	*aph*(3')VIa	Not detected	None	CarO, OmpA, Omp33-36	None	None
**20-0014**	R	R	R	R	R	R	R	R	R	I	OXA-24/40, OXA-51	*aph*(3')VIa, *ant*(2')Ia	Not detected	None	CarO, OmpA, Omp33-36	None	None
**20-0026**	R	R	R	R	R	R	R	R	S	I	OXA-24/40, OXA-51	*aph*(3')VIa	Not detected	None	CarO, OmpA, Omp33-36	None	None
**20-0031**	R	R	R	R	R	R	R	R	S	I	OXA-24/40, OXA-51	*aph*(3')VIa, *ant*(2')Ia	Not detected	AdeABC	None	CarO, OmpA	None
**20-0046**	R	R	R	R	R	R	R	R	S	I	OXA-24/40, OXA-51	*aph*(3')VIa, *ant*(2')Ia	Not detected	AdeFGH	OmpA, Omp33-36	None	None

**20-0048**	R	R	R	R	R	R	R	R	S	I	OXA-24/40, OXA-51	*aph*(3')VIa, *ant*(2')Ia	Not detected	AdeABC, AdeFGH,	Omp33-36	CarO, OmpA	None
**20-0049**	R	R	R	R	R	R	R	R	R	I	OXA-24/40, OXA-51	*aph*(3')VIa	Not detected	AdeABC	Omp33-36	None	Low
**20-0051**	R	R	R	R	R	R	R	R	R	I	OXA-24/40, OXA-51	Not detected	Not detected	AdeABC	CarO, Omp33-36	None	Low
**20-0059**	S	R	R	R	R	R	R	R	R	I	OXA-24/40, OXA-51	Not detected	Not detected	None	OmpA, Omp33-36	None	None
**20-0088**	S	R	R	R	R	R	R	R	R	I	OXA-24/40, OXA-51	Not detected	Not detected	AdeABC	None	CarO, OmpA	None
**20-0098**	S	R	R	R	R	R	R	R	R	I	OXA-24/40, OXA-51	Not detected	Not detected	None	CarO, OmpA, Omp33-36	None	None
**20-0107**	R	R	R	R	R	R	R	R	R	I	OXA-24/40, OXA-51	*aph*(3')VIa, *ant*(2')Ia	Not detected	None	CarO, Omp33-36	None	None
**20-0155**	S	R	R	R	R	R	R	R	R	I	OXA-24/40, OXA-51	*aph*(3')IIa, *aac*(6')Ib	Not detected	AdeIJK	None	None	None
**20-0164**	R	R	R	R	R	R	R	R	S	I	OXA-24/40, OXA-51	*aph*(3')VIa, *ant*(2')Ia	Not detected	AdeABC	Omp33-36	None	None
**20-0194**	S	R	R	R	R	R	R	R	R	I	OXA-24/40, OXA-51	Not detected	Not detected	None	Omp33-36	None	None
**20-0203**	S	R	R	R	R	R	R	R	R	I	OXA-24/40, OXA-51	Not detected	Not detected	AdeABC, AdeIJK	None	None	None
**20-0216**	S	R	R	R	R	R	R	R	R	I	OXA-24/40, OXA-51	Not detected	Not detected	None	CarO, OmpA, Omp33-36	None	None
**20-0230**	S	R	R	R	R	R	R	R	R	I	OXA-24/40, OXA-51	Not detected	Not detected	AdeFGH	Omp33-36	CarO	Low
**20-0232**	S	R	R	R	R	R	R	R	R	I	OXA-24/40, OXA-51	Not detected	Not detected	AdeABC, AdeFGH, AdeIJK	None	CarO, OmpA	None
**20-0237**	S	R	R	R	R	R	R	R	R	I	OXA-24/40, OXA-51	Not detected	Not detected	AdeABC, AdeFGH, AdeIJK	None	CarO, OmpA	High
**20-0291**	R	R	R	R	R	R	R	R	S	I	OXA-24/40, OXA-51	*aph*(3')VIa, *ant*(2')Ia	*parC* mutations	AdeABC	None	CarO, OmpA	None
**20-0329**	S	R	S	R	R	R	S	R	R	R	OXA-24/40, OXA-51	Not detected	Not detected	AdeFGH	None	OmpA, Omp33-36	None
**20-0406**	S	R	R	R	R	R	R	R	R	I	OXA-24/40, OXA-51	Not detected	*parC* mutations	AdeIJK	Omp33-36	None	None
**20-0425**	S	R	R	R	R	R	R	R	S	I	OXA-24/40, OXA-51	Not detected	Not detected	None	Omp33-36	None	None

SAM: ampicillin/sulbactam; TZP: piperacillin/tazobactam; FEP: cefepime; CAZ: ceftazidime; IMI: imipenem, MEM: meropenem;

GEN: gentamicin; LEV: levofloxacin; TGC: tigecycline; COL: colistin; I: intermediate; R: resistant; S: susceptible.
